# Modification of the 4-Hydroxyphenylacetate-3-hydroxylase Substrate Pocket to Increase Activity towards Resveratrol

**DOI:** 10.3390/molecules28145602

**Published:** 2023-07-24

**Authors:** Qianchao Zhang, Yuning Jin, Kai Yang, Sheng Hu, Changjiang Lv, Jun Huang, Jiaqi Mei, Weirui Zhao, Lehe Mei

**Affiliations:** 1 College of Pharmaceutical Science, Zhejiang University of Technology, Hangzhou 310014, China; zqccome@163.com; 2 School of Biological and Chemical Engineering, NingboTech University, Ningbo 315100, China; jinyuning1116@163.com (Y.J.); genegun@zju.edu.cn (S.H.); 3 Department of Chemical and Biological Engineering, Zhejiang University, Hangzhou 310027, China; kayyoung@zju.edu.cn; 4 School of Biological and Chemical Engineering, Zhejiang University of Science and Technology, Hangzhou 310023, China; yangtzelv@zust.edu.cn (C.L.); huangjun@zust.edu.cn (J.H.); 5 Hangzhou Huadong Medicine Group Co., Ltd., Hangzhou 310011, China; meijiaqi@eastchinapharm.com; 6 Jinhua Advanced Research Institute, Jinhua 321019, China

**Keywords:** 4-hydroxyphenylacetate-3-hydroxylase, piceatannol, substrate pocket, semi-rational design, *ortho*-hydroxylation

## Abstract

4-Hydroxyphenylacetate-3-hydroxylase (4HPA3H; EC 1.14.14.9) is a heterodimeric flavin-dependent monooxygenase complex that catalyzes the *ortho*-hydroxylation of resveratrol to produce piceatannol. Piceatannol has various health benefits and valuable applications in food, medicine, and cosmetics. Enhancing the catalytic activity of 4HPA3H toward resveratrol has the potential to benefit piceatannol production. In this study, the critical amino acid residues in the substrate pocket of 4HPA3H that affect its activity toward resveratrol were identified using semi-rational engineering. Two key amino acid sites (I157 and A211) were discovered and the simultaneous “best” mutant I157L/A211D enabled catalytic efficiency (*K*cat/*K*m—resveratrol) to increase by a factor of 4.7-fold. Molecular dynamics simulations indicated that the increased flexibility of the 4HPA3H substrate pocket has the potential to improve the catalytic activity of the enzyme toward resveratrol. On this basis, we produced 3.78 mM piceatannol by using the mutant I157L/A211D whole cells. In this study, we successfully developed a highly active 4HPA3H variant for the hydroxylation of resveratrol to piceatannol.

## 1. Introduction

Piceatannol (3,4,3′,5′-tetrahydroxy-trans-stilbene) is a natural compound containing a catechol motif, initially found in plants [1,2]. Previous studies have suggested that piceatannol possesses anti-cancer [3], anti-obesity [4], and neuroprotective [5] properties. Therefore, piceatannol has a wide range of promising applications in the medical, food, and cosmetic fields [6]. Unfortunately, piceatannol is usually present in low abundance in nature. For example, in grapes, piceatannol is present at a concentration of 0.052 mg/g fresh weight [7], which hampers the exploration and application of its pharmacological properties. Piceatannol can be formed through *ortho*-hydroxylation of resveratrol, which is a relatively inexpensive compound found in many plants at higher concentrations [8] and the catalytic process is regarded as a highly efficient and cost-effective approach for piceatannol production. However, traditional chemical methods are troublesome in facilitating this oxidation reaction, and there is substantial scientific and commercial interest in the development of suitable and efficient enzymes to catalyze the reaction [9,10].

**Figure 1:**
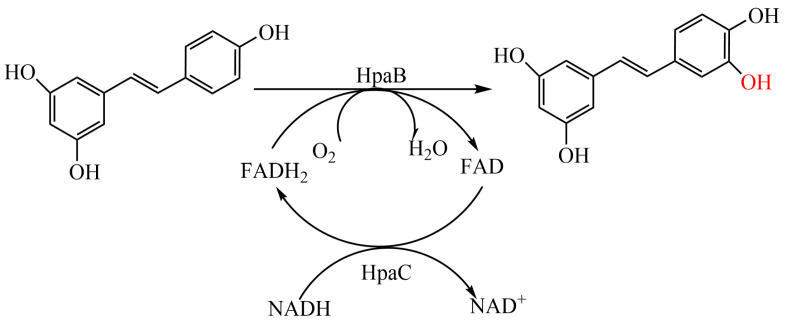
EcHpaBC catalyzes the conversion of resveratrol to piceatannol.

Currently, both cytochrome-P450 hydroxylase (EC 1.14.14.1) and 4-hydroxyphenylacetate-3-hydroxylase (4HPA3H; EC 1.14.14.9) have been reported to synthesize compounds with catechol motifs via *ortho*-hydroxylation of monophenol compounds. In recent decades, P450 hydroxylases have been the dominant group of enzymes that have been explored and engineered for this purpose. However, their application is limited by their low catalytic activity, which is due to the intrinsic catalytic mechanism of P450 enzymes [11,12]. In contrast, 4HPA3H is easily expressed and exhibits high *ortho*-hydroxylation specificity [2,13]. It is a flavin-dependent monooxygenase complex comprising two components [14]. The larger component, flavin-dependent monooxygenase (HpaB), ranges in size from 39 to 63 KDa and is responsible for the *ortho*-hydroxylation of phenolics, determining the substrate profile of 4HPA3H. The smaller component, NAD(P)H-flavin oxidoreductase (HpaC), typically has a molecular weight of 16–35.4 KDa. Its role is to provide FADH2 or FMNH- for monooxygenase (HpaB)-catalyzed reactions by consuming NAD(P)H [15,16]. 4HPA3H specifically catalyzes the ortho-hydroxylation of phenolic compounds and has been discovered in various microorganisms such as *Escherichia coli* [14], *Thermus thermophilus* [17], *Acinetobacter baumannii* [18], *Pseudomonas aeruginosa* [19], and *Pseudomonas putida* [20]. Of these, *E. coli* 4HPA3H (EcHpaBC) accepts a broad spectrum of substrates, including some complex monophenol compounds, and is used to produce hydroxytyrosol [21], naringenin [22], salvianic acid A [23], and piceatannol [9] (Figure 1). However, the catalytic efficiency of EcHpaBC towards resveratrol and some complex monophenol compounds is significantly lower than that of its natural substrate, 4-hydroxyphenylacetic acid (4HPA) [21,22], which severely hinders the use of EcHpaBC in piceatannol production. Therefore, our objective was to enhance the catalytic activity of EcHpaBC towards resveratrol to develop a highly efficient biocatalyst for piceatannol production.

## 2. Results and Discussion

### 2.1. Key Catalytic Amino Acids in the Substrate Pocket

**Figure 2:**
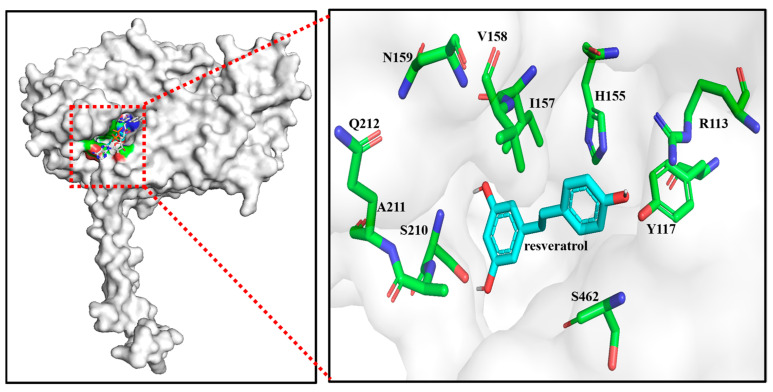
Substrate pocket of EcHpaB binds resveratrol during catalysis.

**Figure 3:**
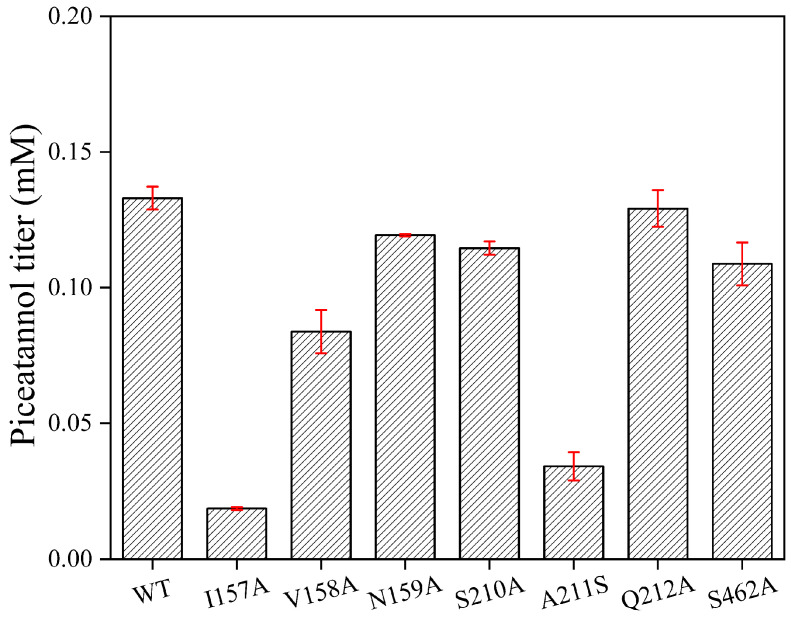
Alanine scan of the EcHpaBC substrate pocket. Reactions were performed at 30 °C, pH 7.4 in reaction mixture comprised of 2 mM resveratrol, 4% dimethyl sulfone, 1% Tween-80, and washed recombinant cells at OD_600_ = 10. Data are presented as mean ± SD of three biological replicates.

Substrate pockets play a crucial role in enzyme catalysis, and the activity of enzymatically catalyzed substrates can be enhanced through targeted modification of amino acid residues within the substrate pocket [24,25,26,27]. To enhance the activity of EcHpaBC toward resveratrol, we used the structure of EcHpaB (PDB:6QYI) to derive a substrate pocket with Autodock-1.5.6 (Figure 2). The substrate pocket comprises 10 amino acid residues: R113, Y117, H155, I157, V158, N159, S210, A211, Q212, and S462, of which R113, Y117, and H155 are catalytic triplets [14]. Subsequently, we performed alanine-scanning mutagenesis to evaluate the effect of substrate pocket sites (I157, V158, N159, S210, A211, Q212, and S462) on the catalytic activity of EcHpaBC towards resveratrol (Figure 3). The results indicated a significant reduction in piceatannol production in the I157A (0.02 mM) and A211S (0.04 mM) mutants compared to that in the WT (0.13 mM), suggesting that I157 and A211 were key sites affecting the catalytic activity of EcHpaBC toward resveratrol.

**Figure 4:**
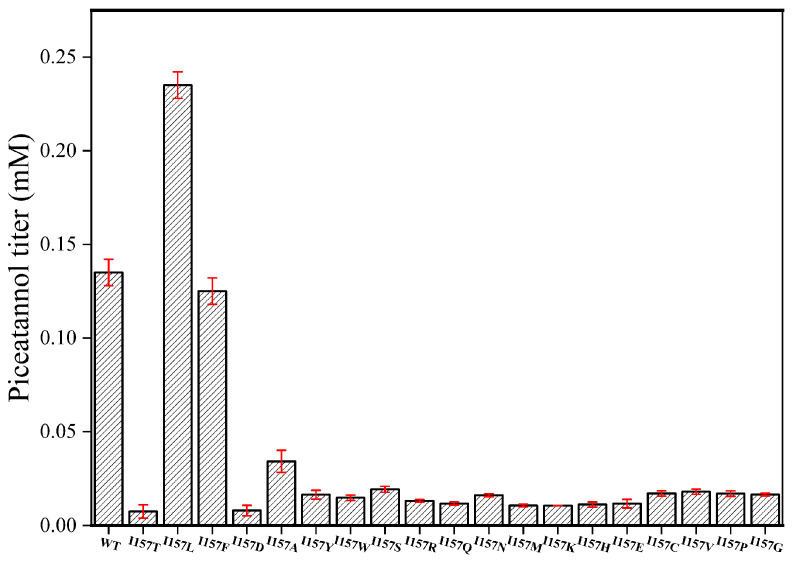
Effect of I157 saturation mutations on the catalysis of resveratrol by EcHpaBC. Reactions were performed at 30 °C, pH 7.4 in reaction mixture comprised of 2 mM resveratrol, 4% dimethyl sulfone, 1% Tween-80, and washed recombinant cells at OD_600_ = 10. Data are presented as mean ± SD of three biological replicates.

**Figure 5:**
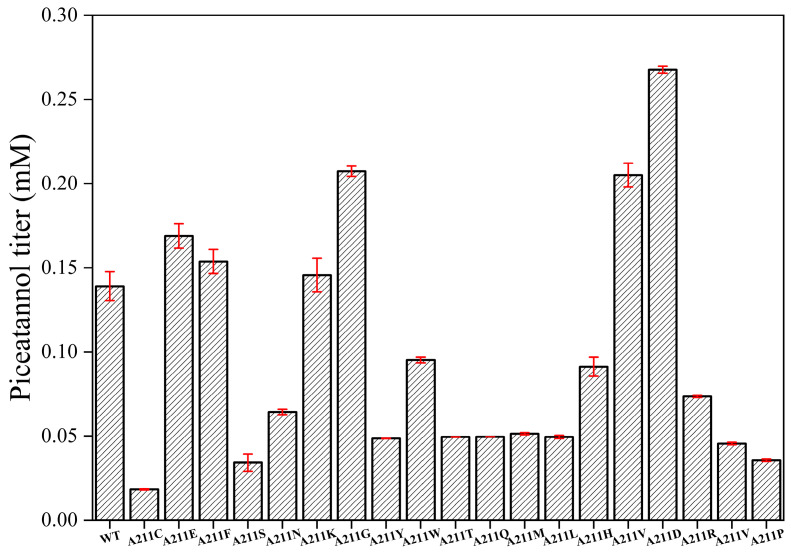
Effect of A211 saturation mutations on the catalysis of resveratrol by EcHpaBC. Reactions were performed at 30 °C, pH 7.4 in reaction mixture comprised of 2 mM resveratrol, 4% dimethyl sulfone, 1% Tween-80, and washed recombinant cells at OD_600_ = 10. Data are presented as mean ± SD of three biological replicates.

**Figure 6:**
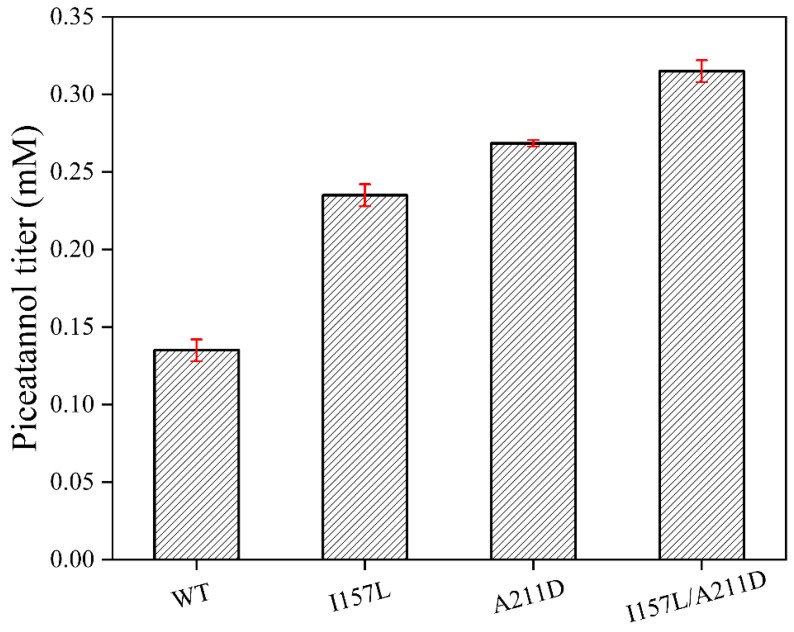
Effect of I157L/A211D on the catalysis of resveratrol by EcHpaBC. Reactions were performed at 30 °C, pH 7.4 in reaction mixture comprised of 2 mM resveratrol, 4% dimethyl sulfone, 1% Tween-80, and washed recombinant cells at OD_600_ = 10. Data are presented as mean ± SD of three biological replicates.

Next, we conducted saturation mutagenesis of I157 and A211 to improve the catalytic activity of EcHpaBC toward resveratrol. Among the I157 mutants, I157L (0.24 mM) displayed the highest catalytic activity towards resveratrol, exhibiting a 1.84-fold increase compared to the WT (Figure 4). Similarly, among the A211 mutants, A211D (0.27 mM) showed a significant improvement, resulting in a 2.07-fold increase in catalytic activity compared to the WT (Figure 5). To further improve the catalytic activity toward resveratrol, we generated a combined I157L/A211D mutant. Remarkably, I157L/A211D (0.32 mM) demonstrated 2.46 times higher resveratrol catalytic activity than the WT (Figure 6).

### 2.2. Determination of Enzymatic Parameters of Mutant Enzymes and Wild-Type (WT)

**Figure 7:**
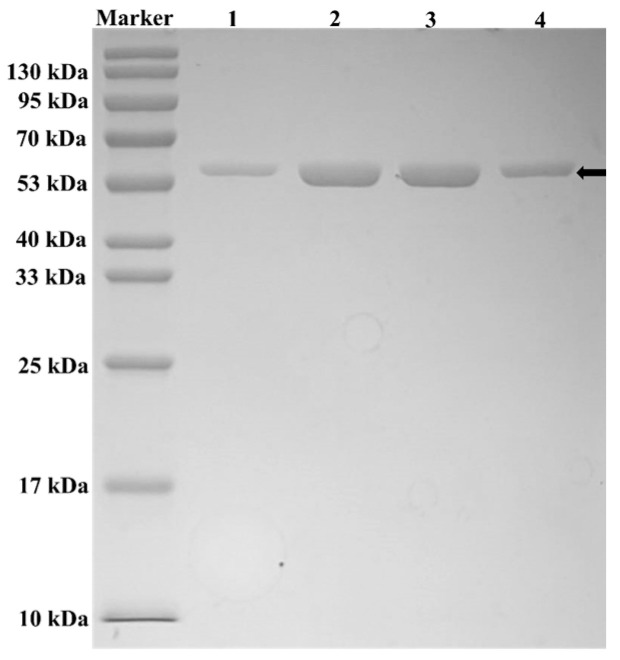
SDS-PAGE analysis of wild-type (WT) and mutant enzymes. Lanes:1 WT, 2 I157L, 3 A211D, 4 I157L/A211D.

To determine the kinetic parameters *K*m and *K*cat for the WT, I157L, A211D, and I157L/A211D mutant enzymes toward resveratrol at 30 °C and pH 7.4, we initially conducted protein purification of WT, I157L, A211D, and I157L/A211D (Figure 7) followed by the determination of their respective kinetic parameters (Table 1). The *K*m values for WT, I157L, A211D, and I157L/A211D were 0.67 mM, 0.33 mM, 0.60 mM, and 1.36 mM, respectively. Interestingly, the I157L/A211D mutant exhibited decreased affinity compared with the WT, whereas the I157L and A211D mutants showed increased affinities relative to the WT. Furthermore, the *K*cat/*K*m values for I157L, A211D, and I157L/A211D were 2.33 min^−1^mM^−1^, 3.15 min^−1^mM^−1^, and 5.72 min^−1^mM^−1^, respectively, representing 1.94-fold, 2.6-fold, and 4.7-fold higher catalytic efficiencies compared to the WT enzyme (1.2 min^−1^mM^−1^). Notably, the I157L/A211D enzyme exhibited the most significant improvement in *K*cat/*K*m relative to the WT, indicating a slightly lower substrate-binding affinity, but distinctly higher catalytic efficiency compared to the WT enzyme. The decrease in I157L/A211D affinity may be due to a lower number of important binding residues and longer hydrogen bonds between HpaB and the substrate (Figures S1 and S2). Conversely, the enlargement of *K*cat may be attributed to an increase in the flexibility of the critical loop region [28]. A similar phenomenon has been observed in the research on molecular modification of glutamic acid decarboxylase [29].

### 2.3. Mechanistic Insights into Enhanced Catalytic Efficiency of Mutants towards Resveratrol

**Figure 8:**
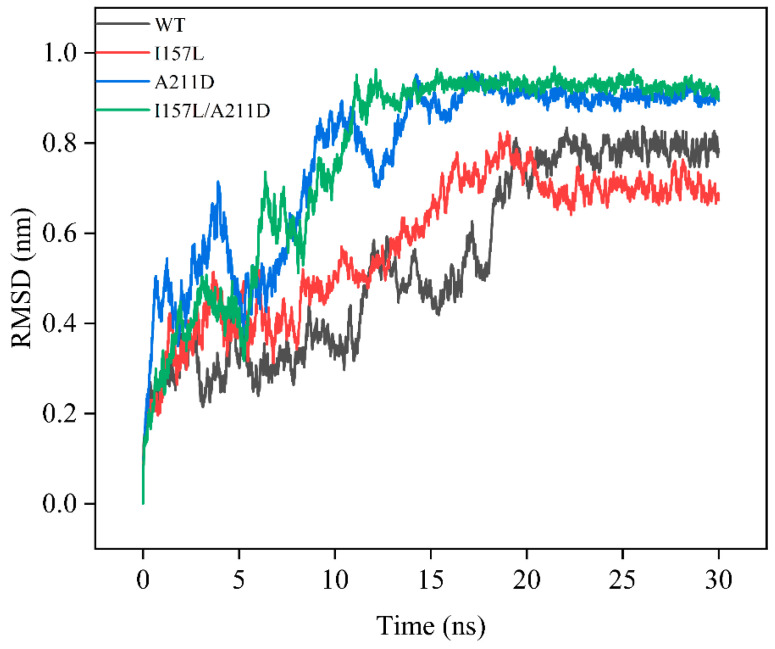
Backbone atom root mean square deviation (RMSD) trajectory analysis of molecular dynamics simulation for the wild-type (WT) and mutant enzymes.

**Figure 9:**
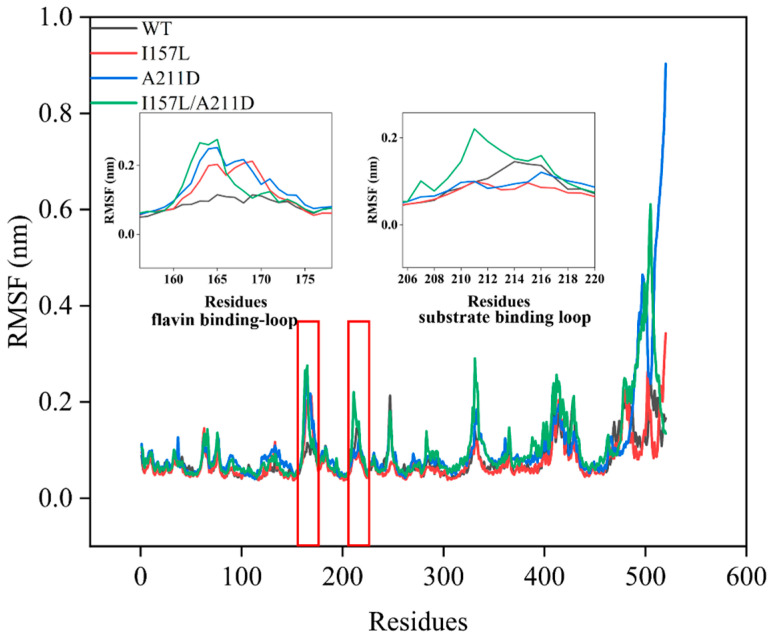
The root mean square fluctuation (RMSF) values of each amino acid calculated from molecular dynamics (MD) simulations.

MD simulations were performed to investigate the mechanism underlying the improved catalytic activity of the I157L, A211D, and I157L/A211D mutants toward resveratrol. The equilibrium state was reached within the last 10 ns of the simulations, as indicated by the root mean square deviation (RMSD) values. At equilibrium, the RMSD values of the WT and mutant enzymes were as follows: 0.80 ± 0.09 nm for the WT, 0.70 ± 0.1 nm for I157L, 0.90 ± 0.030 nm for the A211D, 0.92 ± 0.02 nm for I157L/A211D, (Figure 8). These values suggest that the overall structure of the WT was less flexible than those of A211D and I157L/A211D, but more flexible than that of I157L. The I157 site is located near the flavin-binding loop P161-V171 and is involved in the binding of flavin [14], and the A211 site is situated on the substrate-binding ring G209-E216. Consequently, our focus was directed toward investigating the flexible alterations within the flavin-binding ring P161-V171 and the substrate-binding ring G209-E216. Root mean square fluctuation (RMSF) analysis revealed that the loop residues (P161-V171) exhibited increased flexibility in I157L, A211D, and I157L/A211D compared with the corresponding region in the WT. Additionally, the loop residues (G209-E216) were more flexible in I157L/A211D than in WT. Notably, the flexibilities of I157L and A211D in this region were lower than that of the WT (Figure 9). The overall RMSF of the loop residues (P161-V171) and (G209-E216) near the substrate pockets was higher for the I157L (0.13 nm), A211D (0.15 nm), and I157L/A211D (0.17 nm) mutants than for the WT (0.11 nm). In the past, Shen [22] increased the flexibility of the loop located near the substrate pocket by introducing more flexible amino acids in the G209-E216 loop, effectively increasing the activity of EcHpaBC in catalyzing the complex substrate naringin, which is consistent with our results, indicating that the increased flexibility of the loop near the substrate pocket has the potential to improve the catalytic activity of the enzyme for complex substrates.

### 2.4. Preparation of Piceatannol Using Whole-Cell Catalysts

**Figure 10:**
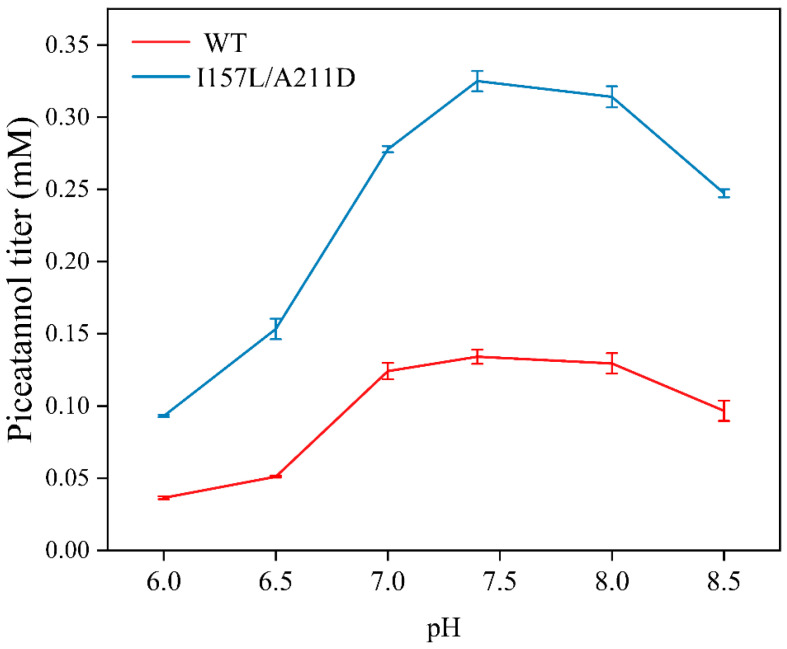
Effects of pH on piceatannol titer. Reactions were performed at 30 °C in reaction mixture comprised of 2 mM resveratrol, 4% dimethyl sulfone, 1% Tween-80, and washed recombinant cells at OD_600_ = 10. Data are presented as mean ± SD of three biological replicates.

**Figure 11:**
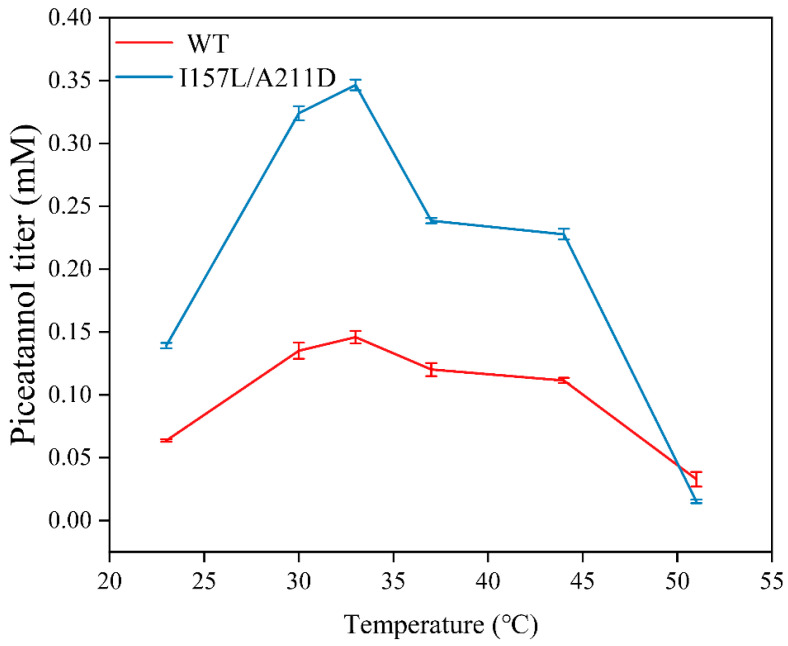
Effects of temperature on piceatannol titer. Reactions were performed at pH 7.4 in reaction mixture comprised of 2 mM resveratrol, 4% dimethyl sulfone, 1% Tween-80, and washed recombinant cells at OD_600_ = 10. Data are presented as mean ± SD of three biological replicates.

We determined the optimal biocatalytic conditions for the WT and I157L/A211D whole cells. The piceatannol production rates of both whole-cell catalysts increased with increasing pH from 6.0 to 7.4 and then decreased at higher pH. The maximal piceatannol conversion rate from resveratrol for both catalysts was obtained at pH 7.4 (Figure 10). The piceatannol yields of both catalysts initially increased with increasing temperature from 16 °C to 33 °C, then decreased with a further increase in temperature above 33 °C (Figure 11). Maximum activity was observed at 33 °C. These results showed that the I157L/A211D mutation did not affect the optimal pH and temperature for EcHpaBC whole cells catalysts.

**Figure 12:**
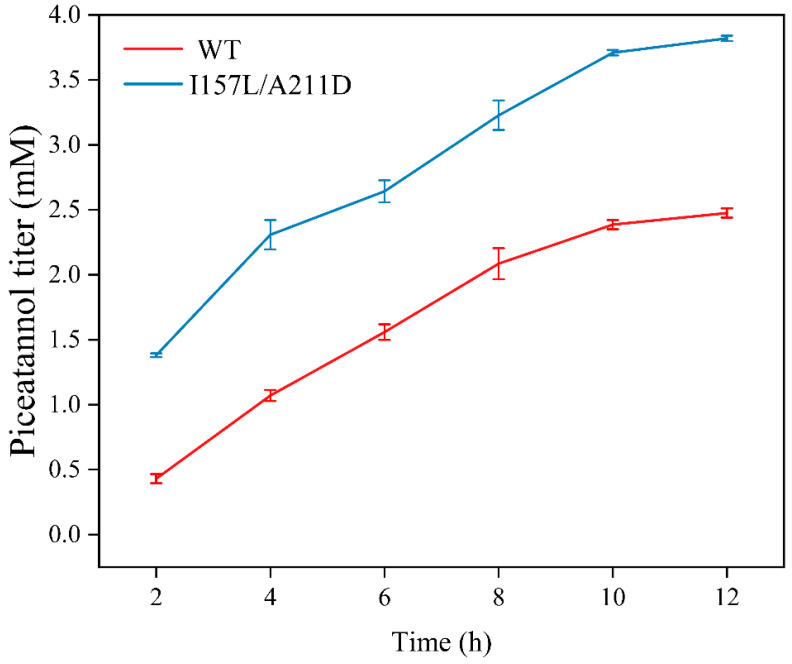
Production curves of piceatannol via whole-cell bioconversion. Reactions were performed at 33 °C, pH 7.4 in reaction mixture comprised of 2 mM resveratrol, 4% dimethyl sulfone, 1% Tween-80, and washed recombinant cells at OD_600_ = 10. And 0.03 mM (0.068 g) resveratrol was added to the 15 mL reaction buffer when the resveratrol concentration was below 0.5 mM. Data are presented as mean ± SD of three biological replicates.

We subsequently performed the biotransformation of resveratrol to piceatannol under optimized conditions. After 12 h, the highest yields achieved were 2.47 mM for WT whole cells and 3.78 mM for I157L/A211D whole cells, respectively. The mutant I157L/A211D whole cells exhibited a 1.53-fold higher piceatannol titer than the WT whole cells. Further increases in the reaction time and resveratrol concentration did not result in an increased piceatannol titer (Figure 12). This may be because high titers of piceatannol inhibit the catalytic activity of HpaBC [30]. 

In this study, we used semi-rational design strategies to improve the activity of EcHpaBC toward resveratrol to broaden the potential for industrial applications of piceatannol. The reasons behind activity improvement were further explored in combination with MD analysis. The *K*cat/*K*m values of mutants I157L, A211D, and I157L/A211D were 1.94-fold, 2.6-fold, and 4.7-fold of WT, respectively, than that of the WT. The increased activity of EcHpaBC toward resveratrol may be due to the increased flexibility of the loop residues (P161-V171) and (G209-E216) near the substrate pocket. Enhanced activity towards complex substrates catalyzed by EcHpaBC and other enzymes, such as glycosyl hydrolase, through modification of the flexibility of the substrate pocket loop has also been observed in previous studies [22,31]. For instance, Shen et al. enhanced the catalytic activity of EcHpaBC towards naringenin by introducing Gly, Ser, and Asp residues on its substrate pocket loop (207–217) and speculated that the increased flexibility of the loop might contribute to the enhanced catalytic activity [22]. Zheng et al. increased the catalytic activity of glycosyl hydrolase toward cytosolic cellotetraose by enhancing the flexibility of the loop in the substrate pocket through modification of the N233 site. Additionally, the movement of the substrate pocket loop in glycosyl hydrolases has been found to facilitate substrate entry and product release [31]. Therefore, modifying the flexibility of the substrate pocket could be an effective approach to improve the catalytic activity of enzymes toward their substrates. 

The efficiency of molecular engineering can be enhanced by predicting crucial amino acid residue sites through alanine scanning of the substrate pockets [32,33,34]. In this study, we performed alanine scans at sites I157, V158, N159, S210, A211, Q212, and S462 located in the substrate pocket and found no significant changes in whole-cell catalytic activity relative to the WT, except for mutants I157A and A211S, which exhibited decreased catalytic activity towards resveratrol. These results indicate that the I157 and A211 sites play crucial roles in catalyzing the conversion of resveratrol to piceatannol. Therefore, we performed saturation mutagenesis of these two sites and found that the whole-cell catalytic activity of resveratrol increased 1.84 and 2.07-fold for mutants I157L and A211D, respectively. Subsequently, we constructed a double mutant, 157L/A211D, and found that its catalytic activity was 2.46-fold higher than that of the WT (Figure 6), with a catalytic synergistic effect. A strategy combining alanine scanning of substrate pockets and saturation mutagenesis of key sites improves the efficiency and feasibility of molecular engineering [34,35,36].

## 3. Materials and Methods

### 3.1. Strains and Materials

*E. coli* BL21(DE3) cells were purchased from Beijing TransGen Biotech Co., Ltd. (Beijing, China). The Mut Express II Fast Mutagenesis Kit V2 was purchased from Nanjing Vazyme Biotech Co., Ltd. (Nanjing, China). Resveratrol and piceatannol were purchased from Yuanye Biotech Co. Ltd. (Shanghai, China). The Ni-NTA Sefinose^TM^ Resin 6FF (Settled Resin) was purchased from Sangon Co., Ltd. (Shanghai, China). Unless otherwise specified, all chemicals were of analytical grade.

### 3.2. Plasmid Construction

For EcHpaBC expression, EcHpaBC (GenBank accession no. CP053602.1) was PCR amplified and inserted into the pETDuet-I plasmid at the *Bam*H I and *Not* I restriction sites, resulting in the pETDuet-EcHpaBC plasmid. Similarly, the EcHpaC gene was PCR-amplified from the *E. coli* genome and cloned into pETDuet-I using *Bam*H I and *Not* I restriction sites to generate the pETDuet-EcHpaC plasmid.

### 3.3. Molecular Docking and Molecular Dynamics Simulations (MD)

Molecular docking was conducted using Autodock-1.5.6, with AutoDock Vina as the docking algorithm. In this study, FAD was assumed to be located near the substrate pockets. The grid box was positioned at the following coordinates: center-x = 113.588, center-y = 6.797, and center-z = −30.8170. The grid spacing parameter was set to 0.375 Å, and the size of the box was 60 × 60 × 60 Å. Twenty docked poses were produced by WT and I157L/A211D (Tables S1 and S2), and the docked pose with a higher affinity score and the phenol group well oriented towards the conserved R113-Y117-H155 triad [14] was selected as the best mode. 

For MD simulations, both WT and mutant enzymes were studied using the Gromacs software (version:2018.4). The OPLS-AA/L all-atom force field was used as the simulated force field. The crystal structure was placed in a cubic box to ensure that the edges of the box were at least 1 nm from the protein surface. The system was solvated with water molecules and Na^+^ ions were added to maintain electrical neutrality. Energy minimization was performed using a 2000-step steepest gradient descent method followed by a 5000-step conjugate gradient method to achieve a reasonable geometric configuration and solvent orientation. Positional confinement was applied during a 100 ps NVP equilibrium and NTP equilibrium to equilibrate the solvent around the protein. Molecular dynamic simulations were conducted for 30 ns at a temperature of 303.15 K.

### 3.4. Construction of Mutants

Site-directed mutagenesis primers were designed based on the EcHpaBC gene sequence. The detailed results are presented in Tables S3–S5. The site-directed PCR amplification was carried out with the following program: initial pre-denaturation at 94 °C for 5 min, followed by denaturation at 94 °C for 30 s, annealing at 60 °C for 30 s, and extension at 72 °C for 5 min, repeated for 30 cycles. Subsequently, the PCR products were treated with *Dpn* I at 37 °C for 1.5 h to eliminate the parental templates. Purified products were transformed into competent *E. coli* BL21(DE3) cells via heat shock. The transformation mixture was plated on LB medium plates supplemented with 100 μg/mL ampicillin and incubated at 37 °C overnight to generate a site-directed mutagenesis library. DNA sequencing was performed by Shanghai Sangon Biotech to verify mutagenesis.

### 3.5. Preparation of the Whole-Cell Biocatalyst

Recombinant *E. coli* cells harboring WT or mutant enzymes were cultured in 100 mL of LB medium supplemented with 100 µg/mL ampicillin at 37 °C with shaking at 200 rpm. Once the optical density at 600 nm (OD_600_) of the culture reached 0.6−0.8, protein expression was induced by adding IPTG (final concentration 0.5 µM), and the culture was continued at 30 °C, 200 rpm for 6 h. Subsequently, the cells were harvested by centrifugation at 6500 rpm and 4 °C for 10 min, followed by two washes with phosphate-buffered saline (PBS).

### 3.6. Whole-Cell Biocatalytic Activity Assay

Whole-cell activity was assessed in a 1 mL reaction solution containing 2 mM resveratrol, 4% dimethyl sulfone, 1% Tween-80, and washed recombinant cells (BL21(DE3)-pETDuet-hpaBC or its mutants) at OD_600_ = 10. Reactions were performed at 800 rpm and 30 °C for 1 h, followed by termination with the addition of 50 µL of 20% hydrochloric acid (HCl). Piceatannol content was analyzed by high-performance liquid chromatography (HPLC).

### 3.7. Enzyme Purification

Induced cells were resuspended in PBS and subjected to ultrasound disruption under the following conditions: 90 cycles of 3 s sonication at 300 W, with 6 s intervals. Centrifugation at 6500 rpm and 4 °C for 10 min was used to remove the precipitated proteins and cellular debris, and the resulting crude enzyme was loaded onto a Ni-NTA affinity column. The column was subsequently washed (washing buffer: 20 mmol·L^−1^ Tris-HCl, 500 mmol·L^−1^ NaCl, 40 mmol·L^−1^ imidazole, pH 7.8) and eluted (elution buffer: 20 mmol·L^−1^ Tris-HCl, 500 mmol·L^−1^ NaCl, 400 mmol·L^−1^ imidazole, pH 7.8). Purified proteins were collected in 2 mL centrifuge tubes.

### 3.8. Enzymatic Parameters of WT Enzyme and Mutant Enzymes

EcHpaB activity assays were conducted as described previously [9], with minor adjustments. A reaction system consisting of 1 mL of PBS, 10 μM FAD, 1 mM NADH, 1 μM HpaB, 1 μM HpaC, and 10–1000 μM substrate was utilized. Reactions involving resveratrol were carried out at 30 °C for 10 min and terminated through acidification with 50 µL of 20% HCl. The reaction rates were determined by measuring product formation and substrate consumption using HPLC. The apparent kinetic parameters were obtained by performing nonlinear regression of the Michaelis–Menten equation using OriginPro 2021.

### 3.9. Optimization of the WT and I157L/A211D-Catalyzed Reactions

To optimize the pH and temperature of the reaction system, the reactions were performed under a range of pH values (6.0–8.5) and temperatures (23–51 °C), using a reaction mixture containing 2 mM resveratrol, 1% *v*/*v* Tween 80, washed recombinant cells (OD_600_ = 10), and 4% *v*/*v* dimethyl sulfoxide. The reactions were conducted at 800 rpm and 30 °C for 1 h and then terminated by adding 50 µL of 20% HCl.

### 3.10. Production of Piceatannol from Resveratrol Using WT and I157L/A211D

To produce piceatannol, biotransformation was conducted using WT and I157L/A211D cells in a 15 mL reaction solution. The solution consisted of 2 mM resveratrol, 1.5 mM ascorbic acid, 4% *v*/*v* dimethyl sulfoxide, 1% *v*/*v* Tween 80, 10% *v*/*v* glycerol, and washed recombinant cells with OD_600_ = 10. The reactions were performed under optimized conditions at an agitation speed of 200 rpm. To address the challenge of dissolving resveratrol in excess of 2 mM, a feeding strategy was implemented for piceatannol mass production. When the resveratrol concentration dropped below 0.5 mM, 0.03 mM (0.068 g) resveratrol was added to 15 mL of reaction buffer. Samples were collected periodically and analyzed using HPLC.

### 3.11. High-Performance Liquid Chromatography (HPLC) Analysis

Resveratrol and piceatannol were quantified using HPLC with a Shimadzu LC-16 system. Piceatannol concentration was determined using an HPLC system equipped with a Supersil ODS2 C18 column (5 μm, 250 × 4.6 mm) and a 210 nm ultraviolet (UV) detector. The mobile phase consisted of water containing 0.05% trifluoroacetic acid (TFA) as solvent A, and acetonitrile containing 0.05% TFA as solvent B. The elution gradient involved a transition from 10% to 70% solvent B over 15 min, followed by a rapid return to 10% solvent B within 1 min, and a final 4 min hold at 10% solvent B. The flow rate was set at 1 mL/min, and the column temperature was maintained at 30 °C.

## 4. Conclusions

In this study, we employed enzyme engineering to obtain mutant enzymes that catalyze higher activity of resveratrol. Among them, the double mutant I157L/A211D enabled a 4.7-fold increase in catalytic efficiency (*K*cat/*K*m—resveratrol). Under optimal reaction temperature and pH, the mutant I157L/A211D whole cells could produce 3.78 mM piceatannol after 12 h. Molecular dynamics simulations indicate that the increased flexibility of the loop near the substrate pocket of 4HPA3H has the potential to improve the catalytic activity of the enzyme for resveratrol. These findings are favorable for the biotransformation of resveratrol and have practical implications for the pharmaceutical industry.

## Figures and Tables

**Table 1:** Kinetic parameters of wild-type (WT) and mutant enzymes.

Name	*K*m (mM)	*K*cat (min^−1^)	*K*cat/*K*m (min^−1^mM^−1^)
WT	0.67 ± 0.12	0.81 ± 0.057	1.2
I157L	0.33 ± 0.056	0.77 ± 0.036	2.33
A211D	0.60 ± 0.030	1.89 ± 0.040	3.15
I157L/A211D	1.36 ± 0.3	7.79 ± 0.35	5.72

## Data Availability

The data presented in this study are available on request from the corresponding author.

## Supplementary Materials

The following supporting information can be downloaded at: https://www.mdpi.com/article/10.3390/molecules28145602/s1, Figure S1: Number and length of hydrogen bonds between WT and resveratrol; Figure S2: Number and length of hydrogen bonds between I157L/A211D and resveratrol; Table S1: Bind poses obtained by docking of WT with resveratrol; Table S2: Bind poses obtained by docking of I157L/A211D with resveratrol; Table S3: Primers used for site-directed mutagenesis; Table S4: Primers used for I157 site-saturating mutation; Table S5: Primers used for A211 site-saturating mutation.

## Sample Availability

Samples of the compounds are not available from the authors.
