# Supplementary material for: Modification of the 4-Hydroxyphenylacetate-3-hydroxylase Substrate Pocket to Increase Activity towards Resveratrol
Source: Molecules. 2023 Jul 24;28(14):5602. doi: 10.3390/molecules28145602 (PMC10384689; doi:10.3390/molecules28145602)
Supplement: Supplementary file 1 [file molecules-28-05602-s001.zip › molecules-2485067-supplementary.pdf]

molecules

MDPI

Supplementary Materials

Table S1. Bind poses obtained by docking of WT with resveratrol. Among them, the configuration highlighted in red is considered the most mode.

mode	Affinity (Kcat/mol)	Rmsd 1.b	Rmsd u.b
1	-7.4	0.000	0.000
2	-7.4	0.100	2.010
3	-7.3	0.163	1.182
4	-7.3	1.468	7.257
5	-7.2	1.402	7.404
6	-7.2	2.527	3.760
7	-7.2	2.449	3.339
8	-7.2	6.289	7.278
9	-7.2	6.300	7.449
10	-7.1	6.226	7.413
11	-7.1	5.578	6.789
12	-7.1	5.565	6.557
13	-7.1	5.852	7.390
14	-7.1	5.570	6.763
15	-7.1	5,567	6.582
16	-7.0	5.644	6.693
17	-6.7	5.852	6.693
18	-6.7	1.868	7.206
19	-6.4	2.931	7.163
20	-6.3	2.928	7.282

Table S2. Bind poses obtained by docking of I157L/A211D with resveratrol. Among them, the configuration highlighted in red is considered the most mode.

mode	Affinity (Kcat/mol)	Rmsd 1.b	Rmsd u.b
1	-6.6	0.000	0.000
2	-6.6	0.015	2.007
3	-6.4	1.636	2.380
4	-6.3	4.488	9.365
5	-6.3	4.489	9.613
6	-6.2	3.671	8.414
7	-6.2	3.679	8.496
8	-6.0	5.762	9.532
9	-6.0	4.801	7.745
10	-5.9	5.816	6.994
11	-5.9	4.750	7.522
12	-5.9	4.833	9.406
13	-5.8	5.367	7.698
14	-5.8	5.990	8.303
15	-5.8	6.308	9.528
16	-5.6	5.664	8.017
17	-5.6	3.463	8.230
18	-5.6	5.528	8.372
19	-5.5	3.004	7.694
20	-5.5	1.873	7.267

Table S3. Primers used for site-directed mutagenesis.

Mutation	Template	Primers (5′-3′)	
I157A	pETDuet-HpaBC	Forward:	TTTAACCACGCGGCAGTTAACCCACCGATCG
		Reverse:	CGGTGGGTTAACTGCCGCGTGGTTAAAGTAG
V158A	pETDuet-HpaBC	Forward:	ACGCGATTGCTAACCCACCG
		Reverse:	GGTGGGTTAGCAATCGCGTG
N159A	pETDuet-HpaBC	Forward:	TTAACCACGCGATTGTTGCCCCACCG
		Reverse:	GACGATCGATCGGTGGGGCAACAATC
S210A	pETDuet-HpaBC	Forward:	ATTGGCTTCGGCGCAGCACAAGTGATGG
		Reverse:	CATCACTTGTGCTGCGCCGAAGCCAATC
A211S	pETDuet-HpaBC	Forward:	TTCGGCTCGGAGTAAGTGATGG
		Reverse:	CATCACTTACTCCGAGCCGAAG
Q212A	pETDuet-HpaBC	Forward:	CTCGGCAGCTGTGATGGGCGAAAAC
		Reverse:	GTTTTCGCCCATCACAGCTGCCGAG
S462A	pETDuet-HpaBC	Forward:	TGAAATCAACTACGCCGGTAGCCAGG
		Reverse:	TCCTGGCTACCGGCGTAGTTGATTTC

Table S4. Primers used for I157 site-saturating mutation.

Mutation	Template	Primers(5′-3′)	
I157V	pETDuet-	Forward:	TAACCACGCGGTTGTTAACCCACCGAT
	HpaBC	Reverse:	ATCGGTGGGTTAACAACCGCGTGGTTA
I157C	pETDuet-	Forward:	TAACCACGCGTGTGTTAACCCACCGAT
	HpaBC	Reverse:	ATCGGTGGGTTAACACACGCGTGGTTA
I157E	pETDuet-	Forward:	TAACCACGCGGAAGTTAACCCACCGAT
	HpaBC	Reverse:	ATCGGTGGGTTAACTTCCGCGTGGTTA
I157F	pETDuet-	Forward:	TAACCACGCGTTCGTTAACCCACCGAT
	HpaBC	Reverse:	ATCGGTGGGTTAACGAACGCGTGGTTA
I157N	pETDuet-	Forward:	TAACCACGCGAATGTTAACCCACCGAT
	HpaBC	Reverse:	ATCGGTGGGTTAACATTCGCGTGGTTA
I157K	pETDuet-	Forward:	TAACCACGCGAAAGTTAACCCACCGAT
	HpaBC	Reverse:	ATCGGTGGGTTAACTTTCGCGTGGTTA
I157T	pETDuet-	Forward:	TAACCACGCGACTGTTAACCCACCGAT
	HpaBC	Reverse:	ATCGGTGGGTTAACAGTCGCGTGGTTA
I157L	pETDuet-	Forward:	TAACCACGCGCTTGTTAACCCACCGAT
	HpaBC	Reverse:	ATCGGTGGGTTAACAAGCGCGTGGTTA
I157R	pETDuet-	Forward:	TAACCACGCGCGTGTTAACCCACCGAT
	HpaBC	Reverse:	ATCGGTGGGTTAACACGCGCGTGGTTA
I157H	pETDuet-	Forward:	TAACCACGCGCATGTTAACCCACCGA-
	HpaBC	Reverse:	TATCGGTGGGTTAACATGCGCGTGGTTA
I157Y	pETDuet-	Forward:	TAACCACGCGTATGTTAACCCACCGAT
	HpaBC	Reverse:	ATCGGTGGGTTAACATACGCGTGGTTA
I157W	pETDuet-	Forward:	TAACCACGCGTGGGTTAACCCACCGAT
	HpaBC	Reverse:	ATCGGTGGGTTAACCCACGCGTGGTTA
I157M	pETDuet-	Forward:	TAACCACGCGATGGTTAACCCACCGAT
	HpaBC	Reverse:	ATCGGTGGGTTAACCATCGCGTGGTTA
I157Q	pETDuet-	Forward:	TAACCACGCGCAAGTTAACCCACCGAT
	HpaBC	Reverse:	ATCGGTGGGTTAACTTGCGCGTGGTTA
I157P	pETDuet-	Forward:	TAACCACGCGCCTGTTAACCCACCGAT
	HpaBC	Reverse:	ATCGGTGGGTTAACAGGCGCGTGGTTA
I157G	pETDuet-	Forward:	TAACCACGCGGGTGTTAACCCACCGAT
	HpaBC	Reverse:	ATCGGTGGGTTAACACCCGCGTGGTTA
I157D	pETDuet-	Forward:	TAACCACGCGGATGTTAACCCACCGAT
	HpaBC	Reverse:	ATCGGTGGGTTAACATCCGCGTGGTTA
I157S	pETDuet-	Forward:	TAACCACGCGTCTGTTAACCCACCGAT
	HpaBC	Reverse:	ATCGGTGGGTTAACAGACGCGTGGTTA

Table S5. Primers used for A211 site-saturating mutation.

Mutation	Template		Primers(5′-3′)
A211V	pETDuet -HpaBC	Forward:	TTGGCTTCGGCTCGGTTCAAGTGATGG
		Reverse:	CCATCACTTGAACCGAGCCGAAGCCAA
A211C	pETDuet -HpaBC	Forward:	TTGGCTTCGGCTCGTGTCAAGTGATGG
		Reverse:	CCATCACTTGACACGAGCCGAAGCCAA
A211E	pETDuet -HpaBC	Forward:	TTGGCTTCGGCTCGGAACAAGTGATGG
		Reverse:	CCATCACTTGTTCCGAGCCGAAGCCAA
A211F	pETDuet -HpaBC	Forward:	TTGGCTTCGGCTCGTTCCAAGTGATGG
		Reverse:	CCATCACTTGGAACGAGCCGAAGCCAA
A211N	pETDuet -HpaBC	Forward:	TTGGCTTCGGCTCGAATCAAGTGATGG
		Reverse:	CCATCACTTGATTCGAGCCGAAGCCAA
A211K	pETDuet -HpaBC	Forward:	TTGGCTTCGGCTCGAAGCAAGTGATGG
		Reverse:	CCATCACTTGCTTCGAGCCGAAGCCAA
A211T	pETDuet -HpaBC	Forward:	TTGGCTTCGGCTCGACTCAAGTGATGG
		Reverse:	CCATCACTTGAGTCGAGCCGAAGCCAA
A211L	pETDuet -HpaBC	Forward:	TTGGCTTCGGCTCGCTTCAAGTGATGG
		Reverse:	CCATCACTTGAAGCGAGCCGAAGCCAA
A211R	pETDuet -HpaBC	Forward:	TTGGCTTCGGCTCGAGACAAGTGATGG
		Reverse:	CCATCACTTGTCTCGAGCCGAAGCCAA
A211H	pETDuet -HpaBC	Forward:	TTGGCTTCGGCTCGCATCAAGTGATGG
		Reverse:	CCATCACTTGATGCGAGCCGAAGCCAA
A211I	pETDuet -HpaBC	Forward:	TTGGCTTCGGCTCGATTCAAGTGATGG
		Reverse:	CCATCACTTGAATCGAGCCGAAGCCAA
A211Y	pETDuet -HpaBC	Forward:	TTGGCTTCGGCTCGTATCAAGTGATGG
		Reverse:	CCATCACTTGATACGAGCCGAAGCCAA
A211W	pETDuet -HpaBC	Forward:	TTGGCTTCGGCTCGTGGCAAGTGATGG
		Reverse:	CCATCACTTGCCACGAGCCGAAGCCAA
A211M	pETDuet -HpaBC	Forward:	TTGGCTTCGGCTCGATGCAAGTGATGG
		Reverse:	CCATCACTTGCATCGAGCCGAAGCCAA
A211Q	pETDuet -HpaBC	Forward:	TTGGCTTCGGCTCGCAACAAGTGATGG
		Reverse:	CCATCACTTGTTGCGAGCCGAAGCCAA
A211P	pETDuet -HpaBC	Forward:	TTGGCTTCGGCTCGCCTCAAGTGATGG
		Reverse:	CCATCACTTGAGGCGAGCCGAAGCCAA
A211G	pETDuet -HpaBC	Forward:	TTGGCTTCGGCTCGGGACAAGTGATGG
		Reverse:	CCATCACTTGTCCCGAGCCGAAGCCAA
A211D	pETDuet -HpaBC	Forward:	TTGGCTTCGGCTCGGATCAAGTGATGG
		Reverse:	CCATCACTTGATCCGAGCCGAAGCCAA

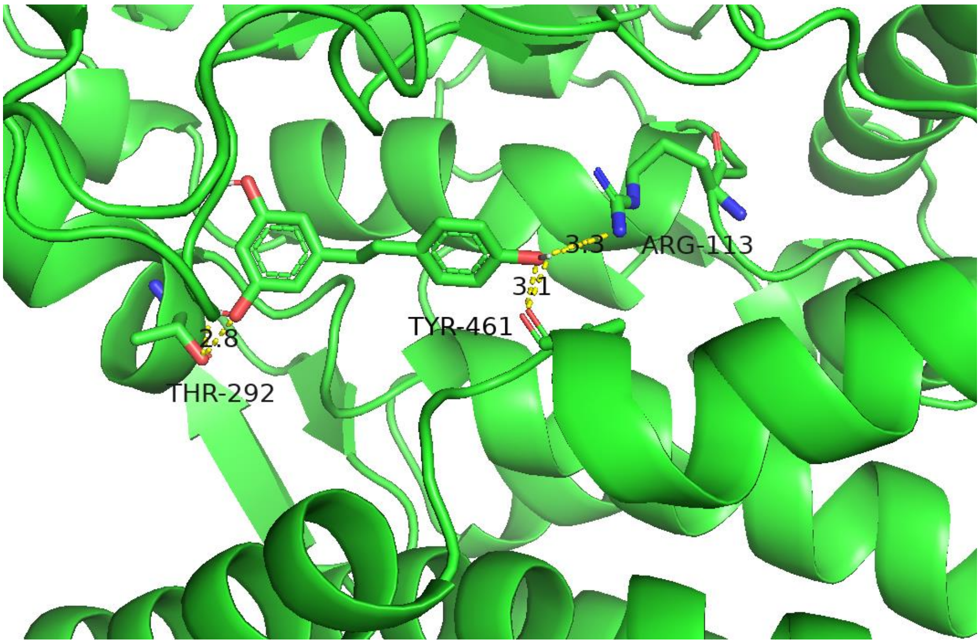

Figure S1. Number and length of hydrogen bonds between WT and resveratrol.

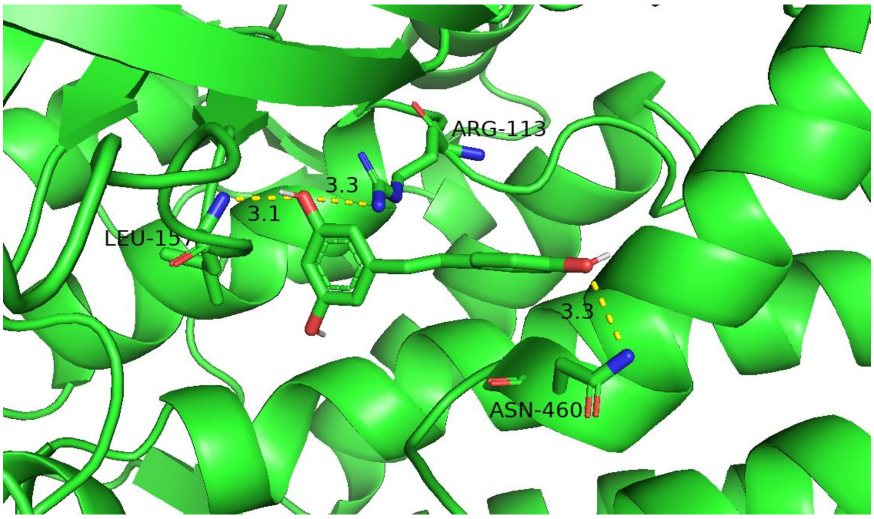

Figure S2. Number and length of hydrogen bonds between I157L/A211D and resveratrol.
